# Supplementary material for: A Screening of Epigenetic Therapeutic Targets for Non-Small Cell Lung Cancer Reveals PADI4 and KDM6B as Promising Candidates
Source: Int J Mol Sci. 2022 Oct 7;23(19):11911. doi: 10.3390/ijms231911911 (PMC9570250; doi:10.3390/ijms231911911)
Supplement: Supplementary file 1 [file ijms-23-11911-s001.zip › ijms-1884091-supplementary.pdf]

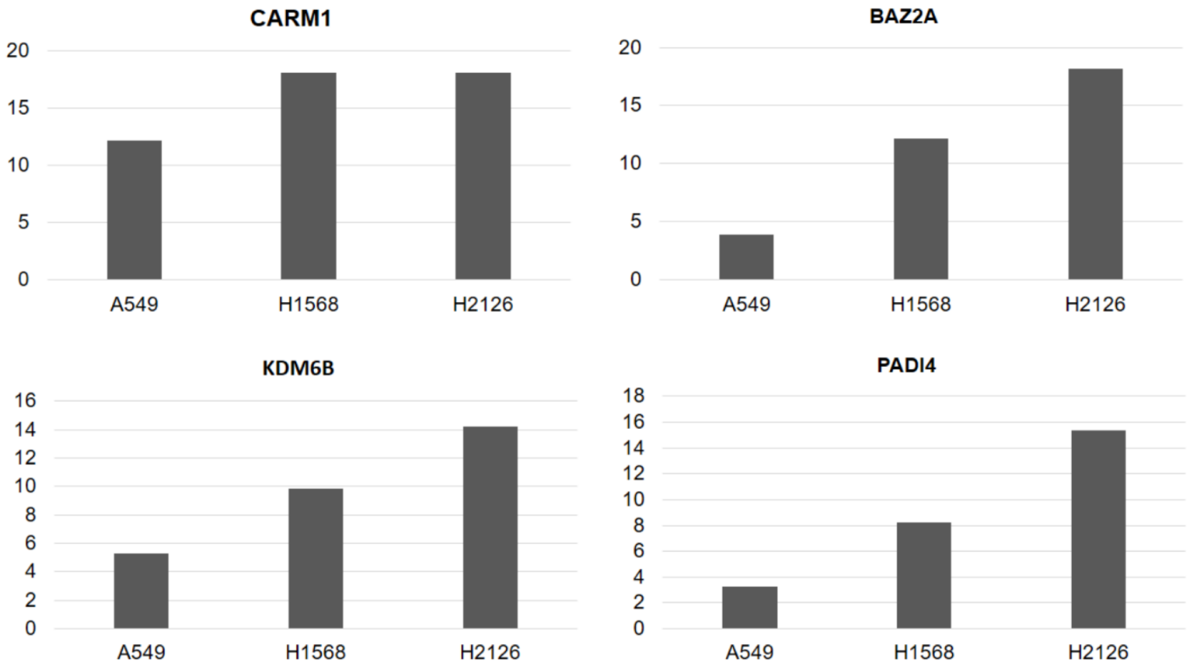

Figure S1. *In silico* gene expression analysis of 4 epigenetic targets in pulmonary adenocarcinoma cell lines. Graphs are presented as the mean of normalized expression values.

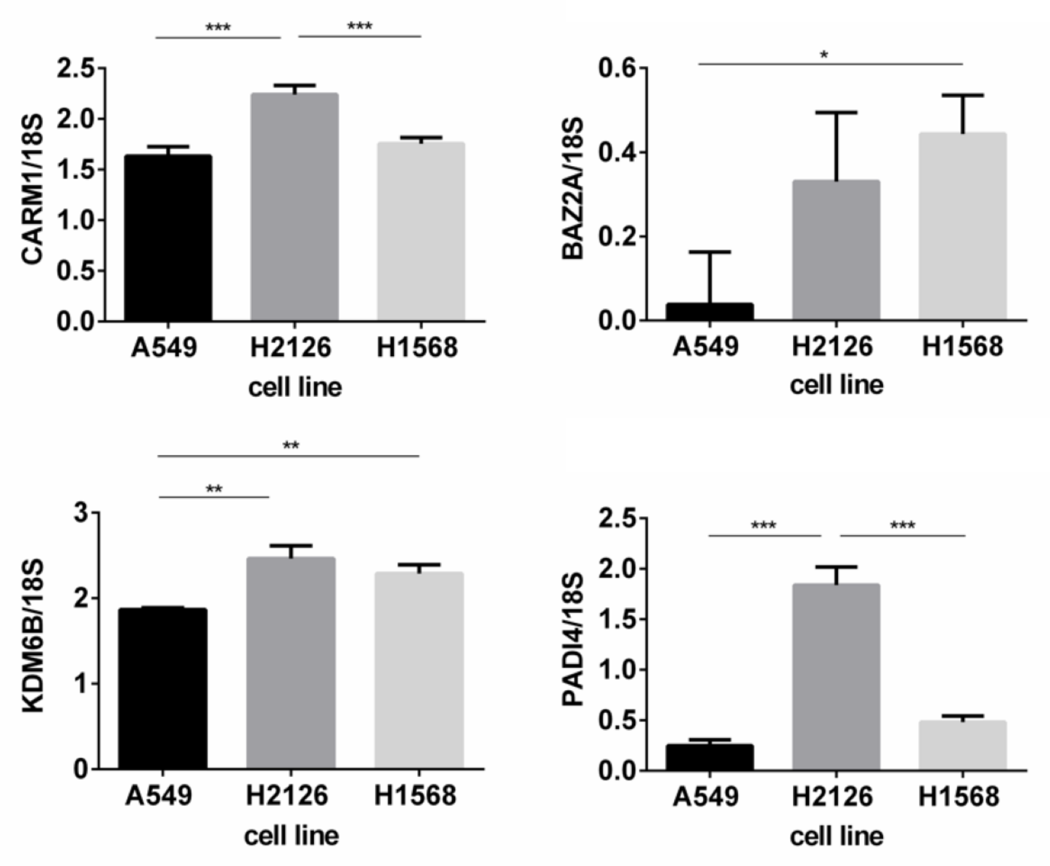

Figure S2. Epigenetic target gene expression (**p* < 0.05; ***p* < 0.01; ****p* < 0.001; *****p* < 0.0001 - One way ANOVA followed by multiple comparisons test from Tukey).

Table S1. Differential gene expression analysis for A549 cell line after the treatment with PADI4 and KDM6B epigenetic inhibitors. The results selected genes based on FDR < 0.05 and LogFC > 1; < -1.

PADI4 (down-regulated genes)				
**Gene**	**logFC**	**logCPM**	**P value**	**FDR**
ABLIM3	-2.16598	3.896844	6.68E-33	2.18E-29
RFTN1	-2.07843	2.583859	1.05E-13	4.42E-11
NIBAN1	-1.74611	4.702511	1.19E-06	7.71E-05
PLEK2	-1.68459	1.889142	1.57E-08	2.16E-06
RYR1	-1.65518	1.805821	6.53E-08	7.12E-06
DHRS2	-1.62446	4.79535	6.82E-16	4.06E-13
UNC5B	-1.5814	2.046634	2.96E-08	3.52E-06
PXDN	-1.57246	1.364956	1.79E-06	0.000109
ATF3	-1.5494	4.000687	4.80E-20	4.46E-17
SERPINA3	-1.47609	1.680433	0.002389	0.02837
RAB39B	-1.44302	2.990452	1.65E-08	2.23E-06
CXCL8	-1.42821	2.971476	2.47E-08	3.06E-06
IL11	-1.41114	2.883052	8.46E-06	0.000386
SYT13	-1.40973	4.231187	1.99E-20	2.00E-17
DRD1	-1.40966	3.293555	2.86E-11	8.70E-09
BARX1	-1.39148	0.826938	0.000908	0.014122
LINC00941	-1.37827	2.190588	2.83E-06	0.000151
KIF1A	-1.35548	1.89791	2.09E-05	0.000803
SNORD83A	-1.34829	0.901237	0.000353	0.007312
SCO2	-1.30857	0.697471	0.002042	0.025266
LYPD1	-1.28858	1.07346	0.00179	0.023015
SNORD63B	-1.28674	0.890429	0.00082	0.013243
NLGN4Y	-1.27874	0.819972	0.00344	0.037143
NCMAP	-1.27447	1.246873	0.000127	0.003378
ZFP3	-1.26491	2.649588	5.98E-07	4.35E-05
NIPAL4	-1.25077	2.722688	1.46E-07	1.31E-05
SNORA64	-1.23225	1.610212	0.000136	0.003553
KCNMA1	-1.21997	4.878998	6.12E-21	7.28E-18
ZSCAN18	-1.21976	1.164495	0.000362	0.007412
HBEGF	-1.21365	1.838905	3.34E-05	0.001181
STC1	-1.19296	0.912192	0.001193	0.017404
KCNH1	-1.18789	3.380463	0.000165	0.004105

BCL2A1	-1.18785	0.883621	0.004105	0.042303
LAMC2	-1.18455	4.414846	1.67E-09	2.84E-07
ZNF542P	-1.18364	1.63012	0.002579	0.030007
EREG	-1.18328	6.424183	3.99E-26	8.71E-23
MIAT	-1.15437	2.792116	1.67E-06	0.000102
SNORA73B	-1.1463	1.702368	8.07E-05	0.002373
TAS2R5	-1.13598	0.88959	0.003545	0.038005
SLC16A6	-1.12895	1.024322	0.004371	0.044111
DGAT2	-1.11783	2.108499	0.000226	0.005254
LINC00471	-1.11283	1.409989	0.000427	0.008256
TRIB3	-1.11014	8.616827	2.38E-05	0.000889
CITED4	-1.10676	3.71473	4.62E-06	0.000229
NCKAP5	-1.09288	2.909523	2.99E-06	0.000158
TNFRSF12A	-1.09209	6.180822	6.35E-15	3.32E-12
FILIP1	-1.08797	2.663604	2.01E-05	0.000776
DMRTA1	-1.08438	1.604028	0.000895	0.014027
LRRC37A6P	-1.07934	1.242138	0.001906	0.024059
DGUOK-AS1	-1.06685	1.644268	0.000254	0.005752
ANKRD1	-1.06572	2.657378	2.29E-06	0.000131
NOS1	-1.05983	1.248634	0.004645	0.045741
ZNF239	-1.05911	2.827497	7.10E-07	5.00E-05
KIRREL3	-1.04891	3.707308	6.74E-10	1.24E-07
SLFNL1	-1.04426	2.31261	0.000421	0.008245
PPP1R15A	-1.03949	7.1728	3.39E-14	1.53E-11
LOC102725231	-1.03552	2.039582	0.000344	0.007163
NAV2	-1.03463	4.876648	3.60E-08	4.17E-06
PABPC1L	-1.01848	6.364791	4.55E-14	1.99E-11
XDH	-1.00992	1.135906	0.005201	0.049397
ZNF420	-1.00923	1.748509	0.000526	0.009627
AKNA	-1.0008	5.240594	2.85E-05	0.001034

**PADI4 (up-regulated genes)**				
**Gene**	**logFC**	**logCPM**	**P value**	**FDR**
FGB	2.570617	6.585985	1.22E-99	1.60E-95
PPARGC1A	2.345716	1.71671	1.19E-10	2.64E-08
SLC16A7	2.111457	3.161757	8.55E-23	1.24E-19
DOCK8	1.884747	1.348758	2.38E-08	3.03E-06
MYOCD	1.845663	1.611589	9.80E-09	1.44E-06
F5	1.767262	3.026287	1.40E-16	8.73E-14
TMEM151A	1.737155	2.251023	6.47E-11	1.63E-08
NRXN3	1.702303	0.867355	4.99E-05	0.001634
ITIH2	1.694332	1.402564	9.44E-08	9.15E-06

NID2	1.688522	1.8109	1.82E-09	2.94E-07
TM4SF4	1.635597	5.034187	8.97E-21	9.79E-18
A1CF	1.633534	2.74931	3.81E-13	1.39E-10
CFH	1.577096	7.470666	1.74E-58	1.14E-54
MUC16	1.52822	5.408622	5.67E-26	1.06E-22
FAXDC2	1.525156	1.018501	4.87E-05	0.001613
CFHR1	1.511834	1.975166	8.11E-08	8.11E-06
KCNJ2	1.501049	1.357311	1.70E-05	0.000679
ADH6	1.494147	1.897476	8.87E-08	8.80E-06
SPTLC3	1.450007	1.662017	6.30E-07	4.55E-05
MROH2A	1.392993	1.010119	0.000121	0.003244
LINC01474	1.392282	0.659862	0.000595	0.010531
LRRC29	1.388649	0.966035	8.24E-05	0.002413
SOX2	1.383915	0.979558	0.000455	0.008675
CHL1	1.37935	0.729878	0.000382	0.007721
HOOK1	1.37373	2.231556	9.18E-07	6.22E-05
GOT1-DT	1.353048	1.383392	0.00044	0.008445
UGT1A7	1.352245	2.208636	6.87E-08	7.23E-06
FGA	1.346355	5.988379	4.64E-33	2.03E-29
TM4SF20	1.334241	6.238076	5.25E-28	1.37E-24
ENTPD2	1.330111	2.454085	2.68E-07	2.11E-05
CEACAM16-AS1	1.327011	0.533268	0.005012	0.048227
STAG3L1	1.321903	0.513889	0.003226	0.035388
TM4SF5	1.321381	0.829997	0.000782	0.012869
ASB4	1.320708	2.539827	3.16E-06	0.000166
KCNJ16	1.320141	1.168848	0.001778	0.022919
KCNQ1	1.313789	0.727692	0.000572	0.010216
DPEP1	1.312026	2.063608	1.30E-06	8.24E-05
TENM3	1.309977	4.936187	4.28E-25	7.00E-22
DDIT4L	1.303926	1.90864	6.49E-06	0.000303
CEACAM1	1.303799	3.445515	4.59E-11	1.28E-08
TNFRSF13C	1.281089	1.668056	0.000149	0.003785
NPY1R	1.27707	1.034076	0.000558	0.010053
LOC105377744	1.276757	0.772785	0.001068	0.016102
RNF224	1.274336	0.983359	0.000293	0.006386
NEB	1.263852	3.473212	2.50E-08	3.06E-06
UNC13D	1.260612	4.64725	1.33E-16	8.69E-14
LOC101926887	1.258073	1.008998	0.002482	0.029194
CDH16	1.254207	0.84291	0.001268	0.018054
COL1A1	1.248641	3.175263	1.60E-09	2.76E-07
ZNF554	1.229245	1.240794	0.000205	0.00489
BCAS1	1.221997	4.132543	1.79E-10	3.85E-08

IFIT1	1.178667	1.761142	0.000228	0.00529
ARHGEF16	1.174711	3.00665	2.23E-07	1.86E-05
NRIP3	1.170341	0.95106	0.001669	0.021906
ANK3	1.150479	2.475505	3.94E-05	0.001356
PHEX	1.148861	1.189953	0.000452	0.008644
SH2B2	1.148361	3.474898	1.70E-10	3.71E-08
UPK3B	1.146103	1.910386	7.17E-05	0.002173
FLRT2	1.145093	1.445011	0.000172	0.004253
KCTD13-DT	1.131037	1.473627	0.000131	0.003437
PCAT7	1.126435	1.122828	0.001979	0.024672
CORO2A	1.125714	4.747707	1.01E-18	8.28E-16
ARID5B	1.105971	3.272508	6.83E-08	7.23E-06
CIDEC	1.104077	1.379261	0.000362	0.007412
S1PR5	1.09904	1.482257	0.00126	0.018019
RORA	1.093546	0.839522	0.002436	0.028766
KCNQ1OT1	1.092971	1.895612	7.84E-05	0.002321
KLF9	1.089679	3.878726	6.37E-10	1.19E-07
VIL1	1.087472	1.070202	0.002196	0.026522
FBXO2	1.084228	2.155773	6.36E-05	0.001973
VASH2	1.082665	2.676476	2.68E-06	0.000146
GPR199P	1.079519	0.964713	0.003169	0.034947
TJP3	1.074671	3.837274	3.42E-11	9.94E-09
QPRT	1.068836	3.531338	5.90E-10	1.12E-07
KRT4	1.066978	4.705169	2.42E-13	9.60E-11
FGG	1.06431	5.238643	5.11E-20	4.46E-17
C1orf21	1.052845	1.703124	0.000165	0.004105
MYO1D	1.043369	2.764934	1.28E-06	8.16E-05
BCL2L15	1.042217	1.243708	0.003053	0.034062
LOC107985728	1.040762	4.791001	1.37E-12	4.61E-10
LOC105373424	1.03886	1.63829	0.003362	0.036572
FAM83A	1.037041	1.849497	0.000478	0.008969
ALDOC	1.033297	1.424643	0.003119	0.03461
SKIDA1	1.031406	1.393316	0.000675	0.011569
PDK4	1.028369	7.009559	2.62E-18	2.01E-15
DCDC1	1.023172	1.854705	0.000833	0.013374
NPNT	1.020227	3.10865	4.66E-07	3.45E-05
EIF2AK3-DT	1.018657	0.910279	0.004377	0.044134
LIN7A	1.011893	4.496597	3.58E-13	1.34E-10
ATP6V0D1-DT	1.004192	1.334714	0.002926	0.033018

KDM6B (down-regulated genes)

Gene	logFC	logCPM	P value	FDR
PRDM1	-1.00918	2.289796	3.07E-05	0.000457
ANKRD1	-1.01703	2.662901	2.39E-06	4.89E-05
KIF1A	-1.01714	1.979568	0.003475	0.022257
MYPN	-1.03131	0.811733	0.008964	0.045505
HK2	-1.03778	1.076437	0.004464	0.026911
ZNF583	-1.05113	1.023525	0.001577	0.012154
KCNMA1	-1.05279	4.928109	1.12E-29	6.05E-27
RFTN1	-1.06209	2.805277	1.24E-07	3.67E-06
KLC2-AS1	-1.0629	0.872893	0.003692	0.023349
ZSCAN18	-1.07563	1.184041	0.000509	0.004875
SNORA73B	-1.07615	1.707718	4.21E-05	0.000605
ZNF561-AS1	-1.07662	0.97798	0.001467	0.011502
MSS51	-1.07841	1.101731	0.001592	0.012255
ZFP3	-1.09129	2.68986	1.09E-07	3.27E-06
ZFP82	-1.09731	1.561737	0.000181	0.002067
MATN1-AS1	-1.10297	1.143075	0.001105	0.009121
LOC112268035	-1.10403	0.862505	0.00171	0.012948
FUT1	-1.10483	1.335725	0.000239	0.002597
CDCP1	-1.13683	3.919086	2.12E-15	2.15E-13
ZNF239	-1.13949	2.797196	4.06E-09	1.68E-07
LOC112268412	-1.14073	1.084645	0.000505	0.004847
SCO2	-1.15653	0.715384	0.002841	0.019163
LAMP3	-1.16051	0.247896	0.010156	0.049873
SLCO2B1	-1.16589	1.126291	0.001799	0.013444
ATF3	-1.18165	4.09472	8.54E-18	1.21E-15
SYT13	-1.18472	4.290846	1.23E-17	1.73E-15
THBD	-1.1887	2.697802	1.19E-06	2.65E-05
FBXW10	-1.18972	2.398985	1.32E-06	2.89E-05
ACY1	-1.19254	0.737035	0.001834	0.01364
FILIP1	-1.20499	2.622781	1.90E-06	3.97E-05
ABI3BP	-1.21655	0.05122	0.008286	0.043073
ZDHHC14	-1.2228	1.543091	0.00025	0.002696
MSX1	-1.22676	0.801199	0.00179	0.013418
SLIT1	-1.23374	0.550856	0.0019	0.014031
ADGRE1	-1.23723	0.4614	0.003585	0.022823
RAB39B	-1.24426	3.033895	2.07E-07	5.84E-06
CXCL8	-1.26235	3.005777	2.57E-10	1.33E-08
DHRS2	-1.28607	4.881452	6.15E-20	1.20E-17
EGLN2	-1.29528	1.498515	0.000137	0.001622
SLC2A1-DT	-1.31344	0.051177	0.007315	0.039311
HBEGF	-1.31694	1.798411	7.91E-07	1.84E-05

LINC00638	-1.3413	0.575132	0.002282	0.016197
EXOC3L2	-1.37088	0.736812	0.000228	0.002496
HNRNPR	-1.51659	0.119557	0.001237	0.010007
PLAC1	-1.59695	0.611281	8.53E-05	0.001099
SLC16A6	-1.67655	0.871747	3.47E-05	0.000509
NOS1	-1.68312	1.082928	2.83E-05	0.000427
ZNF420	-1.6859	1.566053	2.33E-08	8.23E-07
DMGDH	-1.78063	0.307399	0.000319	0.003305
TTC41P	-1.85169	0.032922	0.000185	0.002109
ABLIM3	-1.86819	3.948763	4.34E-33	3.26E-30
BARX1	-1.87199	0.703033	1.42E-05	0.000236
NLGN4Y	-1.97709	0.657243	6.57E-06	0.000121
SUSD5	-2.15096	0.433536	4.78E-06	9.08E-05
PXDN	-2.21284	1.228312	8.45E-10	3.99E-08
CIART	-2.7148	-0.37603	1.02E-05	0.000178

Source: Lesbon, J. C. C. (2022).

Table S2. Inhibitory epigenetic molecules acquired in collaboration with Structural Genomics Consortium (SGC).

Epigenetic target	Enzyme class	Probe
BRD2	Bromodomains	JQ1
BRD3	Bromodomains	JQ1
BRD4	Bromodomains	JQ1
BRDT	Bromodomains	JQ1
BAZ2B	Bromodomains	GSK2801/BAZ2-ICR
BAZ2A	Bromodomains	GSK2801/BAZ2-ICR
BRPF1	Bromodomains	NI-57
BRD1	Bromodomains	NI-57
BRPF3	Bromodomains	NI-57
SMARCA2	Bromodomains	PFI-3
SMARCA4	Bromodomains	PFI-3
PBRM1	Bromodomains	PFI-3
SMYD2	Metiltransferases	BAY-598
KDM6B	Demetilases	GSK-J4
ADA2	Bromodomais	NVS-CECR2-1
CECR2	Bromodomais	NVS-CECR2-2
PADI4	Deiminases	GSK484
DOT1L	Metiltransferases	SGC0946
WDR5	Metiltransferases	OICR-9429
SETD7	Metiltransferases	R-PFI-2

IDH1	IDH1 mutant inhibitor	GSK864
EZH2	Metiltransferases	GSK343
KDM1A	Demetilases	GSK-LSD1
L3MBTL3	Reader de lisinas metiladas	UNC1215
BRD9	Bromodomains	Bi-9564
BRD7	Bromodomains	Bi-9564
EZH1	Metiltransferases	UNC1215
KMT5B	Metiltransferases	A-196
KMT5C	Metiltransferases	A-196
EHMT2	Metiltransferases	A-366
CREBBP	Acetilases	SGC-CBP30
EP300	Acetilases	SGC-CBP30
PRMT1	Metiltransferases	MS023
PRMT3	Metiltransferases	MS023
CARM1	Metiltransferases	TP-064/MS023
PRMT5	Metiltransferases	MS023
PRMT6	Metiltransferases	MS023
PRMT8	Metiltransferases	MS023

Source: Lesbon, J. C. C. (2022).

**KDM6B (up-regulated genes)**				
**Gene**	**logFC**	**logCPM**	**P value**	**FDR**
LOC100507634	2.91893	-0.17269	8.84E-08	2.74E-06
TMEM132D-AS1	2.55070	1.53118	5.74E-14	4.70E-12
LOC105373180	2.49009	-0.49535	1.73E-05	0.00027748
FGB	2.42660	6.45541	2.99E-266	4.04E-262
TMED2-DT	2.38016	-0.30233	1.22E-05	0.00020591
HS3ST3B1	2.30133	0.15776	4.56E-07	1.15E-05
ADGRV1	2.20827	0.00157	1.06E-05	0.00018409
SLC16A7	2.18723	3.21911	1.47E-37	1.53E-34
F13B	2.15555	0.28386	1.99E-06	4.14E-05
BCHE	2.10230	0.23827	0.0007448	0.00671062
MYOCD	2.08913	1.81244	3.62E-14	3.06E-12
F5	2.02463	3.22218	3.36E-32	2.27E-29
CTTNBP2	2.00077	1.03086	1.23E-08	4.69E-07
BCO2	1.92912	0.25348	0.00013704	0.00162185
TEX19	1.88915	-0.13298	0.00033765	0.00347018
DOCK8	1.87354	1.35195	3.65E-09	1.53E-07
VIL1	1.86738	1.62090	3.33E-11	2.04E-09
LOC101927699	1.82739	0.31321	2.75E-05	0.00041889

LOC102724908	1.78606	0.65342	5.15E-06	9.70E-05
LOC107986087	1.77341	-0.21533	0.00049751	0.00478227
ACSL5	1.75182	0.07265	0.00011311	0.00137474
LOC105377744	1.68256	1.04906	6.21E-06	0.00011457
LOC105378539	1.66500	-0.37244	0.00234837	0.01658213
IDI2-AS1	1.62544	-0.15317	0.00154094	0.01195511
SMAD9	1.62518	0.71061	0.0002376	0.00258752
TM4SF4	1.61606	5.00907	1.92E-51	5.20E-48
KCNJ16	1.58668	1.34369	2.45E-06	4.99E-05
MROH2A	1.57543	1.13476	1.12E-06	2.49E-05
LOC105370941	1.56653	-0.19430	0.00197322	0.01446989
ADH6	1.56473	1.95099	2.22E-10	1.16E-08
FAM110B	1.56337	0.86866	9.71E-06	0.00017036
LOC105376506	1.53950	0.38498	0.00089339	0.00777975
CFH	1.51705	7.42085	1.21E-149	8.21E-146
CHL1	1.48821	0.81750	5.29E-05	0.00072601
LOC105377685	1.47954	0.25193	0.00069772	0.00634569
BMPR1B-DT	1.47433	0.43973	0.00046858	0.00455277
CFHR1	1.47310	1.95477	1.34E-08	5.03E-07
A1CF	1.47047	2.63674	3.22E-14	2.74E-12
DCDC1	1.44488	2.14591	2.05E-09	9.05E-08
PPARGC1A	1.43509	1.03928	1.18E-05	0.00020147
DNAJC3-DT	1.40363	0.08976	0.00222498	0.01591038
LGI4	1.39660	0.34184	0.00511923	0.02998355
CCNE2	1.39523	0.34174	0.00095873	0.00820598
ENG	1.38111	0.81702	0.000215	0.00238953
RTKN2	1.37257	1.79983	1.41E-07	4.12E-06
ABR	1.36219	-0.05483	0.0047209	0.02813807
LAD1	1.35047	0.68705	0.00021264	0.0023672
LOC105374868	1.34845	0.26725	0.00209957	0.01528041
LOC105371419	1.34113	0.15696	0.00436661	0.02645217
LOC102724378	1.33918	0.05435	0.00404719	0.02496662
LINC01389	1.33174	-0.13322	0.00529932	0.0307316
PRR15L	1.32871	0.74299	0.00107163	0.00895615
NID2	1.32283	1.56492	1.50E-06	3.25E-05
TENM3	1.32271	4.93870	1.27E-42	2.46E-39
CALML6	1.32026	-0.01767	0.00391348	0.02436238
COLCA1	1.31428	0.58137	0.00695685	0.03794263
MIR29B2CHG	1.30943	1.09971	9.75E-05	0.00122629

BCL2L15	1.30728	1.40779	4.53E-05	0.00064199
ZNF608	1.30548	1.88240	6.61E-06	0.00012121
C6orf201	1.30452	0.38352	0.00839665	0.04335283
SOX2	1.30061	0.91817	0.00030358	0.00317071
SPTLC3	1.28716	1.56399	5.09E-06	9.62E-05
ENTPD2	1.28382	2.42463	2.50E-08	8.67E-07
SEPTIN3	1.27438	0.31230	0.00266797	0.01817422
FGA	1.27204	5.92917	8.47E-55	2.86E-51
PAX7	1.27155	3.93623	3.68E-22	1.01E-19
NRXN3	1.26894	0.60433	0.00457801	0.02748636
SHH	1.26680	3.05432	8.53E-10	4.01E-08
UNC13D	1.26412	4.64299	9.43E-26	3.75E-23
NRTN	1.25603	0.76442	0.00080912	0.00717534
SEC24B-AS1	1.24466	0.72096	0.00151821	0.0118278
COL1A1	1.24446	3.17070	1.89E-09	8.41E-08
STAG3L1	1.24439	0.46580	0.00275711	0.01865908
ITIH2	1.24337	1.10832	0.0001004	0.00125644
MUC16	1.23856	5.19335	1.73E-29	8.99E-27
LOC105370092	1.23185	0.72001	0.00149433	0.01169415
ANXA8	1.23078	1.65457	2.98E-06	5.93E-05
INHA	1.22899	-0.07435	0.0078922	0.0417305
LOC102724030	1.22665	0.03674	0.00877312	0.04475979
SOX21-AS1	1.22348	0.53074	0.00140991	0.01114975
FLJ16779	1.21312	1.90104	6.52E-07	1.56E-05
UGT1A7	1.21281	2.12009	1.20E-07	3.57E-06
LOC100506990_1	1.19371	0.12306	0.00861269	0.04411504
TM4SF20	1.19082	6.13098	1.82E-50	4.11E-47
ENTPD8	1.18210	0.58041	0.00174691	0.0131823
LOC107985000	1.17830	0.46564	0.00259243	0.01783543
LOC105373449	1.17364	0.15660	0.00826702	0.04298916
LOC105376505	1.17040	0.75244	0.00656816	0.03644036
HES2	1.16912	1.02996	0.00276148	0.01866068
CCDC39	1.16847	1.46737	9.25E-05	0.00117375
SH2B2	1.16345	3.48421	2.90E-14	2.50E-12
KCTD13-DT	1.16325	1.50051	1.70E-05	0.00027402
HNMT	1.15876	0.64002	0.00505764	0.02973206
MYO1D	1.15479	2.84093	2.77E-09	1.18E-07
ASPDH	1.14225	0.55562	0.00236215	0.01662731
CIDEC	1.13750	1.40058	0.00061523	0.00572646

MIATNB	1.13275	1.18992	0.00177363	0.01331704
LOC105373195	1.13146	1.53314	0.00012194	0.00146621
LOC107985728	1.12502	4.84150	6.84E-22	1.85E-19
LOC105369308	1.12494	0.39725	0.0088978	0.04524218
FGG	1.12300	5.27165	8.02E-32	4.93E-29
SNORD87	1.12204	0.46593	0.00561186	0.03226046
KCNT2	1.11135	2.38230	2.35E-07	6.43E-06
SCX	1.11058	0.80582	0.0085579	0.04397722
EPHA5	1.11047	0.91729	0.0013245	0.01057333
BCAS1	1.10178	4.04358	3.10E-13	2.36E-11
UPK3B	1.09826	1.88529	2.92E-05	0.0004379
TMEM151A	1.09460	1.80913	1.66E-05	0.00026901
GPR199P	1.09131	0.98394	0.00129707	0.01039735
NEB	1.08543	3.34670	1.66E-09	7.42E-08
LOC100130027	1.08285	1.59439	4.66E-05	0.00065553
PRG4	1.08132	1.74381	0.00020193	0.00227761
ADGRG5	1.07911	0.32629	0.00913474	0.04613452
PDZD3	1.07822	0.47883	0.00632528	0.035442
RND1	1.07811	4.48559	5.20E-19	9.12E-17
BTNL9	1.07759	3.00116	8.81E-08	2.74E-06
LINC02747	1.07558	1.28383	0.00103141	0.00869589
LOC105377743	1.06443	1.07302	0.00102693	0.0086721
WHAMMP4	1.06112	0.79636	0.0059365	0.03386736
ASB4	1.05791	2.35983	1.43E-06	3.10E-05
PLXNC1	1.04132	0.92704	0.00249928	0.01730418
LOC102723961	1.04029	1.62333	0.00151199	0.01179827
GGT5	1.03605	2.40541	3.53E-06	6.90E-05
LOC107984421	1.03445	0.49158	0.00778925	0.04138041
ELMO1	1.03194	3.80243	2.52E-14	2.21E-12
GLIPR1L1	1.02675	0.55544	0.00697232	0.03801167
MYO1A	1.02611	2.62836	1.77E-06	3.71E-05
OVGP1	1.01537	1.90420	7.02E-05	0.00092966
ARID5B	1.01418	3.20809	4.14E-08	1.39E-06
CD82	1.01401	2.32026	2.46E-06	5.01E-05
CABCOCO1	1.01294	0.90785	0.00455071	0.02734673
AFF1-AS1	1.01114	0.96449	0.00246386	0.01715559
SYNDIG1	1.01054	1.19860	0.00253554	0.01751935
LOC107987266	1.00975	0.74164	0.00521019	0.03037141

Table S3. Primer sequence of epigenetic targets evaluated by RT-qPCR.

Gene	Sequence (5′-3′)
CARM1 fw	CACACCGACTTCAAGGACAA
CARM1 rev	AAAAACGACAGGATCCCAGA
BAZ2A fw	AAGATGTGTGGCTACAATGG
BAZ2A rev	TCTGCACCCATCAGCTCCG
KDM6B fw	GCCTCTTCTCCACCAAGACC
KDM6B rev	GCCTGGTACTGTGCGTACTT
PADI4 fw	GGCAAAGTGAAGCCAACCAG
PADI4 rev	GTCACAGTTCACCAGCAGGA

Source: Lesbon, J. C. C. (2022).
